# Poor Prestroke Glycemic Control Increases the Rate of Symptomatic Intracranial Hemorrhage after Mechanical Thrombectomy

**DOI:** 10.3390/jcm13051227

**Published:** 2024-02-21

**Authors:** Piotr Luchowski, Maciej Szmygin, Elzbieta Barton, Katarzyna Prus, Hanna Szmygin, Krzysztof Pyra, Remigiusz Ficek, Konrad Rejdak

**Affiliations:** 1 Department of Neurology and Neurological Nursing, Medical University of Lublin, 20-954 Lublin, Poland; elzbieta.barton@umlub.pl; 2 Department of Interventional Radiology and Neuroradiology, Medical University of Lublin, 20-954 Lublin, Poland; maciejszmygin@umlub.pl (M.S.); krzysztofpyra@umlub.pl (K.P.); 3 Department of Neurology, Medical University of Lublin, 20-954 Lublin, Poland; katarzynaprus@umlub.pl (K.P.); remek.ficek@gmail.com (R.F.); konradrejdak@umlub.pl (K.R.); 4 Department of Endocrinology, Center of Oncology of the Lublin Region St. Jana z Dukli, 20-090 Lublin, Poland; koszelh@gmail.com

**Keywords:** diabetes, thrombectomy, ischemic stroke, glycosylated hemoglobin

## Abstract

(1) **Background**: Diabetes is a well-established risk factor for acute ischemic stroke (AIS). This study evaluated the impact of prestroke glycemic control in diabetic patients on their 3-month clinical outcome after mechanical thrombectomy (MT). (2) **Methods**: AIS patients with a premorbid modified Rankin scale (mRS) score of 0–2 who were admitted within 6 h after stroke onset and treated with MT between January 2020 and August 2023 were retrospectively analyzed. The study evaluated the effect of prestroke glycemic control on the stroke severity, reperfusion rate, symptomatic intracranial hemorrhage (sICH) and favorable clinical outcome (modified Rankin scale score 0–2) at 3 months after endovascular treatment. (3) **Results**: A total of 364 patients were analyzed, with 275 cases of non-diabetes (ND), 66 of well-controlled diabetes (WCD) and 23 of poorly controlled diabetes (PCD). There was no significant difference in the baseline neurological deficit expressed according to the National Institutes of Health Stroke Scale among the three groups. The time from stroke onset to groin puncture was similar in the ND, WCD and PCD groups (median 215 min, 194.5 min and 222.5 min, respectively). There was no significant difference in the favorable 3-month clinical outcomes among these three groups (35.2% of ND patients, 42.4% of WCD patients and 39.1% of PCD patients) or full recovery (12.4% of ND patients, 11.0% of WCD patients and 17.4% of PCD patients). The rate of sICH was significantly higher in the PCD group as compared to the ND and WDP groups (21.7% of PCD patients versus 7.6% of ND patients, *p* = 0.038, and 6.0% of WCD patients, *p* = 0.046), but the 3-month mortality did not differ between the three groups (21.8% of ND group, 19.7% of WCD group and 26.1% of PCD group). (4) **Conclusions**: This study shows that poor prestroke glycemic control in AIS diabetic patients does not change the chance of a good clinical functional outcome after endovascular treatment. However, the increased risk of hemorrhagic complications in this group of patients should be considered.

## 1. Introduction

Acute ischemic stroke (AIS) is a leading cause of death and disability [1]. Its treatment should be focused on immediate reperfusion to reestablish proper blood flow. Among patients with acute large cerebral vessel occlusion, mechanical thrombectomy (MT) alone or combined with intravenous thrombolysis (IVT) demonstrated favorable functional outcomes in several clinical trials and was incorporated into daily clinical practice [2].

Diabetes mellitus (DM) is a well-established independent but modifiable risk factor for stroke, both ischemic and hemorrhagic [3]. Approximately 20–30% of AIS patients have diabetes, with most of them having type 2. Many previous studies showed an association between comorbid diabetes and increased stroke severity, decreased survival rate, increased length of hospital stay and poorer functional outcomes after stroke. Recently, a few studies evaluating the effect of acute hyperglycemia on the clinical outcome of AIS patients treated with MT were published [4,5]. These studies reported that a high glucose level at admission as well as a high fasting glucose level after endovascular treatment were associated with unfavorable functional outcomes at 3 months after MT. It should be noted that hyperglycemia in AIS is commonly observed not only in patients with diabetes but has also been reported in non-diabetic patients [6]. Moreover, some studies show that the presence of acute hyperglycemia in non-diabetic AIS patients may predict an increased risk of in-hospital mortality and hemorrhagic transformation and an overall increased risk of poor functional recovery [7,8]. Compared with random glucose measurement, glycosylated hemoglobin (HbA1c) testing has the advantage of providing an average measure of glycemia over the past 90 days, indicating diabetes control and management.

A number of studies have assessed the glucose levels at different time points in relation to stroke onset and treatment. Given that glucose levels are easily modifiable, it may be difficult and error-prone to properly evaluate the effect of glucose levels (at a given time point) on the outcome of endovascular treatment. Thus, the aim of this study was to investigate the effect of long-time prestroke glycemic control on the stroke severity, reperfusion rate, symptomatic intracranial hemorrhage (sICH) and favorable clinical outcome (modified Rankin scale score of 0–2) at 3 months after endovascular treatment in AIS patients.

## 2. Materials and Methods

In this monocentric study, we retrospectively analyzed the prospectively collected data of 364 consecutive patients admitted with acute ischemic stroke due to large vessel occlusion (LVO) who were treated with endovascular thrombectomy between January 2020 and August 2023. One group consisted of patients without a history of DM (non-diabetes, ND) and the second group included patients with DM. Further, diabetic patients were divided into patients with well-controlled prestroke diabetes (WCD group; HbA1c level < 7%) and patients with poorly controlled diabetes (PCD group; HbA1c level ≥ 7%). HbA1c was tested on admission in all patients, regardless of their history of diabetes.

The following inclusion criteria were adopted: (1) ischemic stroke due to LVO confirmed according to imaging examination (non-contrast CT and CT angio and/or MRI); (2) time from symptom onset to reperfusion within 6 h; (3) a National Institute of Health Stroke Scale (NIHSS) score ≥ 6; (4) a prestroke Rankin Scale (mRS) score ≤ 2. The exclusion criteria included (1) the presence of intracranial hemorrhage (ICH) on a baseline CT; (2) a lack of integral laboratory data; (3) no follow-up.

The demographic, clinical and laboratory results on admission as well as time metrics were collected and evaluated. In accordance with the current guidelines of Polish Neurological Society for the Management of Patients with Ischemic Stroke, intravenous thrombolysis (rt-PA) was administered if patients arrived in a time window < 4.5 h in the case of no contraindication [9]. An institutional review committee (IRB) approved this study—approval number KE-0254/285/2019. All procedures were conducted in accordance with the Declaration of Helsinki.

### 2.1. Endovascular Treatment

All procedures were performed by an experienced (at least 5 years of interventional radiology training) radiologist under a biplane angiography unit using 3D rotational angiography. Depending on the patients’ status, conscious sedation or general anesthesia was performed. Mechanical thrombectomy was carried out with aspiration, a stent retriever or a combination of both techniques according to the operator’s preference and experience. Final recanalization was assessed according to the Thrombolysis in Cerebral Infarction (mTICI) classification. Good recanalization was defined as TICI 2b and TICI 3. Complications related to the procedure were noted.

### 2.2. Follow-Up

A routine non-contrast brain CT was performed within the first 24 h following the procedure in order to evaluate brain infarction and assess the occurrence of intracranial hemorrhage (ICH). ICH was classified as symptomatic according to the classification of the European-Australasian Acute Stroke Study (ECASS II) [10]. The clinical outcome was assessed based on the mRS score 90 days after the procedure. A favorable result was defined as an mRS score ≤ 2. The mortality rate was noted.

### 2.3. Statistical Analysis

The statistical analysis was conducted using the StatSoft Statistica 13.1PL package. The patients were classified into three groups, ND, WCD and PCD, as stated above, and comparisons were made on the demographic data, initial NIHSS, risk factors, use of IVT, procedural details and clinical outcome. Student’s *t*-test, the Mann–Whitney test and Chi-squared Pearson’s tests were used when appropriate. Statistical significance was defined as *p* ≤ 0.05.

## 3. Results

During the study period (from January 2020 to August 2023), 364 AIS patients treated by endovascular means were analyzed: 275 ND, 66 WCD and 23 PDC.

There were almost no significant differences among the three groups as far as the baseline clinical characteristics and initial neurological deficits expressed according to the NIHSS scale were concerned (Table 1). The occurrence of peripheral artery disease was statistically more frequent in the group of PCD compared to the ND group (*p* = 0.01). The PCD and WCD groups did not differ in this respect. The most common risk factors included hypertension (47.2% of ND patients, 54.57% of WCD patients and 56.5% of PCD patients), atrial fibrillation (21.1% of ND patients, 25.8% of WCD patients and 34.8% of PCD patients) and hyperlipidemia (31.1% of ND patients, 34.8% of WCD patients and 39.1% of PCD patients) and did not differ significantly among the analyzed groups.

The incidence of vessel occlusion was similar with regard to the internal carotid artery (18.2% of ND patients, 16.7% of WCD patients and 13.0% of PCD patients), the horizontal segment of the MCA (M1) and Sylvian (M2) segment (62.9% of ND patients, 68.1% of WCD patients and 65.2% of PCD patients) and the basilar artery/posterior cerebral artery (6.2% of ND patients, 4.6% of WCD patients and 4.3% of PCD patients). However, tandem occlusions (defined as the occlusion of the internal carotid artery and unilateral occlusion of the middle cerebral artery) were more frequent in the PCD patients compared to patients with no history of DM (*p* = 0.02). The occurrence of tandem occlusion did not differ between PCD and WCD (*p* = 0.28).

The times from door to picture, door to groin and onset to groin were comparable among the three groups (Table 2). The endovascular procedure time was also similar in all the analyzed groups (76.0 ± 41.2 min for ND, 69.8 ± 34.6 min for WCD and 68.5 ± 32.3 min for PCD patients) (Table 2). The successful recanalization rate (mTICI 2b-3) as well as the lack of a good recanalization rate (mTICI 0-2a) appeared similar in the three groups (79.6% for ND patients versus 81.8% for WCD patients and 86.9% for PCD patients for TICI 2b-3 and 20.4% for ND patients versus 18.2% for WCD patients and 13.1% for PCD for TICI 0-2a).

### 3.1. Clinical Outcome

All patients were evaluated using the NIHSS 24 h after the procedure and at discharge. The average NIHSS scores after 24 h and at discharge were similar in the three analyzed groups (8.4 in ND group, 8.1 in WCD group and 9.2 in PCD group 24 h after MT and 7.5 in ND group, 8.0 in WCD group and 8.4 in PCD group at discharge). The mean hospitalization time was 11.8 days (from 1 to 60 days) for the ND patients and 11.4 (from 2 to 49 days) for the WCD patients. The mean hospitalization time for the PCD patients was prolonged to an average of 14.3 days (from 2 to 52 days) (PCD vs. ND, *p* < 0.05; PCD vs. WCD, *p* < 0.05). In 29 (10.5%) of the ND patients, 8 (12.1%) of the WCD patients and 4 (17.4%) of the PCD patients, hematoma at the site of the arterial puncture occurred, with 1 patient from the ND group requiring surgical intervention.

Symptomatic ICH was similar in the ND and WCD groups (Table 2). However, the rate of sICH was significantly higher in the PCD group as compared to the ND and WDP groups (21.7% of PCD patients versus 7.6% of ND patients, *p* = 0.038, and 6.0% of WCD patients, *p* = 0.046).

**Figure 1:**
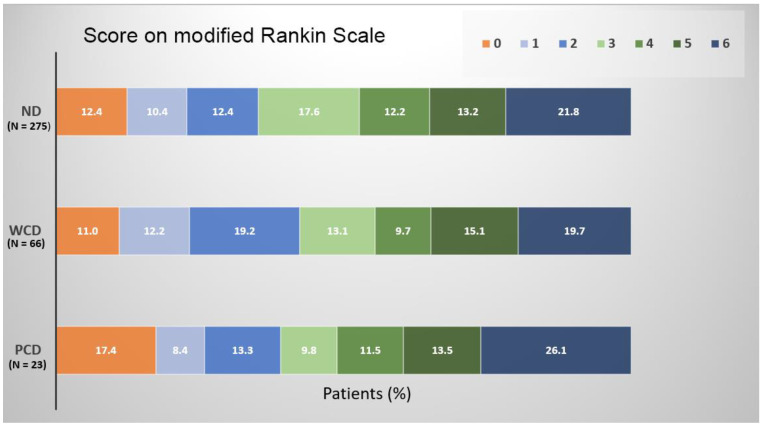
Score on modified Rankin scale.

There was no significant difference in the favorable 3-month clinical outcomes between these three groups (35.2% of ND patients, 42.4% of WCD patients and 39.1% of PCD patients) or full recovery (12.4% of ND patients, 11.0% of WCD patients and 17.4% of PCD patients). Similarly, the rate of 3-month mortality was comparable in the three studied groups (21.8% of ND group, 19.7% of WCD group and 26.1% of PCD group) (Figure 1).

### 3.2. Prognostic Factors

A baseline comparison of the three groups showed no significant difference in the NIHSS on admission and in the procedural details (door-to-picture time, door-to-groin time and endovascular procedure time). However, there was a statistically significant difference in the rate of sICH and the length of hospitalization between the PCD patients and the other two studied groups, i.e., the ND and WCD patients. Taking the three groups together, an HbA1c level ≥ 8.4% was a statistically significant predictor of sICH and a prolonged stay in hospital compared to an HbA1c level < 8.4% (*p* = 0.042 and *p* = 0.035, respectively; Chi-squared Pearson’s test).

## 4. Discussion

The impact of glycemia on ischemic stroke is a topic of significant importance in the field of stroke management. Several studies have shown that the glucose levels at the time of stroke onset can influence the severity and outcomes of AIS. The elevation of plasma glucose levels may be induced by stress hyperglycemia, a marker of the activation of the hypothalamic–pituitary–adrenal axis. There is no unified definition of stress hyperglycemia, and patients are generally classified as diabetic and acute-disease-related hyperglycemic [11]. Stress hyperglycemia, commonly observed not only in diabetic patients, is demonstrated to have a strong relationship with a high risk of adverse outcomes not only in AIS but also in several acute cerebrovascular diseases. For example, stress hyperglycemia in non-diabetic patients with myocardial infarction undergoing primary percutaneous coronary intervention was associated with increased cardiogenic shock and higher mortality [12]. Similarly, in non-diabetic patients, acute stress hyperglycemia was positively correlated with the severity of stroke and a poor neurological outcome [8]. Stress hyperglycemia was also associated with a significantly increased risk of a poor three-month outcome and three-month mortality and symptomatic intracranial hemorrhage in non-diabetic AIS patients undergoing thrombolytic therapy [13].

On the other hand, the association between acute hyperglycemia and the outcomes of AIS patients with diabetes mellitus is controversial. As mentioned previously, several published data regarding stroke and hyperglycemia demonstrated that stress hyperglycemia in non-diabetic patients was associated with an increased risk of mortality after stroke, but surprisingly this was not observed in patients with a history of diabetes [6,14]. This phenomenon was also confirmed in other critical diseases. Acute hyperglycemia with pre-existing diabetes mellitus led to lower mortality and a shorter length of intensive care unit stay compared to patients without diabetes [14,15]. Newly published studies support this observation that, in contrast to non-diabetic patients, there is no clear association between acute hyperglycemia and a worse clinical outcome after AIS among diabetic patients. Merlino et al. recently reported that severe stress hyperglycemia was associated with significantly increased functional dependency, mortality and hemorrhagic complications in AIS patients only when they were not affected by diabetes [16]. However, there are also some studies showing contrary results. A large retrospective study involving over one hundred and sixty thousand AIS patients demonstrated a significantly higher in-hospital mortality among diabetic patients with stress-induced hyperglycemia, with an odds ratio of 1.8 (95% confidence interval 1.10–2.92), as compared to non-diabetic patients [17]. Further analyses showed that stress hyperglycemia had a better predictive value for mortality than that of fasting blood glucose [17].

Only a few studies have investigated the relationship between the glucose levels and clinical outcome in AIS patients after MT. Most of them found an independent association between stress hyperglycemia and a lower rate of excellent outcomes and a higher mortality risk [13,18,19]. This effect was observed independently of the background of hyperglycemia and concerned patients with diabetes and without diabetes [20]. In contrast to stress hyperglycemia, HbA1c reflects the average blood glucose levels during the previous 3 months, providing a stable and reliable indicator of long-term blood glucose control. Lower HbA1c levels generally indicate better blood glucose control, while higher levels suggest poorer control and an increased risk of cardiovascular complications. The American Diabetes Association provides guidelines for target HbA1c levels of less than 7%. Less stringent targets (such as an HbA1c level of 7.5–8%) might be considered for individuals with a history of severe hypoglycemia, limited life expectancy, advanced complications or significant comorbid conditions. To our knowledge, there has been no study exploring different premorbid levels of diabetes control on the clinical outcomes in patients with AIS undergoing MT.

This study found no significant difference between the degree of diabetic control and a favorable 3-month clinical outcome after MT. Patients with poorly controlled diabetes had a similar chance of recovery to patients with well-controlled diabetes and patients without diabetes. Similarly, the rate of mortality was comparable among the three studied groups, with some tendency toward higher mortality in poorly controlled diabetes. However, the rate of sICH was significantly higher in the poorly controlled diabetic patients compared to the non-diabetic as well as properly controlled diabetic patients. A higher rate of sICH in poorly controlled diabetic patients was associated with a prolonged hospitalization time.

Several mechanisms may play a role in the observed association between background hyperglycemia and a higher rate of intracranial hemorrhage after MT. Firstly, prolonged hyperglycemia causes endothelial dysfunction in humans and animals. This can lead to the inhibition of vasodilation via the stimulation of thromboxane A2 release and the downregulation of endothelial nitric oxide synthase expression [21]. The impairment of nitric oxide production, the most valid endogenous vasodilator, is responsible for an increase in systemic vascular resistance. Secondly, prolonged hyperglycemia leads to a pro-inflammatory and pro-thrombotic phenotype augmenting reperfusion injury after successful recanalization. During reperfusion, oxygen-rich blood returns to the ischemic tissue, providing abundant oxygen substrate for the production of vascular superoxide [22]. Interestingly, reactive oxygen species production increases in both normoglycemic and hyperglycemic animals, though the increase is more dramatic in hyperglycemic animals [23]. The depletion of nicotinamide adenine dinucleotide phosphate (NADPH) and inadequate glutathione levels, two powerful antioxidants, may render hyperglycemic vasculature particularly susceptible to peroxynitrite’s damaging effects. Thirdly, the overproduction of reactive oxygen species and perhaps peroxynitrite may promote matrix metalloproteinase 9 (MMP-9) activation and, this way, increase the risk of hemorrhage. MMP-9 is a zinc endopeptidase that degrades components of the extracellular matrix. In ex vivo studies, endothelial cells exposed to prolonged hyperglycemic conditions showed elevated MMP-9 activity, which could be ameliorated by free radical scavengers [23,24]. Moreover, elevated MMP-9 levels have been associated with hemorrhagic conversion after r-tPA or mechanical thrombectomy treatment in stroke patients [25,26,27]. Thus, in AIS patients with poorly controlled diabetes, their increased risk of symptomatic hemorrhagic transformation might be explained in part by increased MMP-9 activity, though further studies are needed to confirm this hypothesis and understand its relationships with the time course and mechanisms of effect.

There were several limitations to our study. Firstly, HbA1c is proportional to the mean blood glucose during the twelve weeks before the test, and its level is a useful indicator of long-term blood glucose control. The condition of blood glucose control during hospitalization was not under consideration. Future studies should evaluate the blood glucose levels at multiple time points and explore the relationship between poorly controlled diabetes with dynamic blood glucose changes after stroke onset and its impact on clinical outcome in AIS patients treated with MT. Secondly, due to the retrospective observational design of the study and the small size of the population, the type of diabetes was not distinguished, and a cause–effect relationship could not be confirmed. Moreover, despite a clearly higher percentage of patients with secondary hemorrhage in the poorly controlled diabetes group, it did not have a statistically significant impact on the clinical outcome three months after mechanical thrombectomy. This effect may be observed after increasing the sample size.

## 5. Conclusions

In conclusion, poor prestroke glycemic control in AIS diabetic patients does not change the chance of a good clinical functional outcome after endovascular treatment. Based on this study and the literature, it can be suggested that in patients with diabetes treated endovascularly, peri-procedure glycemia has a significantly greater impact on the effect of thrombectomy than the degree of diabetes control before the stroke onset. However, the increased risk of hemorrhagic complications in this group of patients should be carefully considered.

## Figures and Tables

**Table 1:** Demographic aspects of the study population.

Characteristic	Non-Diabetic Group (ND) (*n* = 275)	Well-Controlled Diabetic Group (WCD) (*n* = 66)	Poorly Controlled Diabetic Group (PCD) (*n* = 23)	*p* Value
Baseline Males (*n*, %)	123 (44.7%)	34 (51.5%)	11 (47.8%)	0.32 ND vs. WCD ^c^ 0.77 ND vs. PCD ^c^ 0.76 WCD vs. PCD ^c^
Age, y (mean)	71.8 ± 11.95	76.68 ± 11.25	74.55 ±11.08	0.24 ND vs. WCD ^a^ 0.34 ND vs. PCD ^a^ 0.54 WCD vs. PCD ^a^
Baseline NIHSS	17.4 ± 5.5	18.3 ± 5.6	18.0 ± 5.9	0.57 ND vs. WCD ^b^ 0.78 ND vs. PCD ^b^ 0.89 WCD vs. PCD ^b^
IV thrombolysis	206 (74.9%)	54 (81.8%)	14 (60.9%)	0.24 ND vs. WCD ^c^ 0.22 ND vs. PCD ^c^ 0.08 WCD vs. PCD ^c^
Risk factors (*n*, %) Hypertension	130 (47.2%)	36 (54.5%)	13 (56.5%)	0.28 ND vs. WCD ^c^ 0.39 ND vs. PCD ^c^ 0.87 WCD vs. PCD ^c^
Atrial fibrillation	58 (21.1%)	17 (25.8%)	8 (34.8%)	0.41 ND vs. WCD ^c^ 0.13 ND vs. PCD ^c^ 0.41 WCD vs. PCD ^c^
Hyperlipidemia	91 (33.1%)	23 (34.8%)	9 (39.1%)	0.78 ND vs. WCD ^c^ 0.56 ND vs. PCD ^c^ 0.71 WCD vs. PCD ^c^
PAD	74 (26.9%)	21 (31.8%)	12 (52.2%)	0.42 ND vs. WCD ^c^ **0.01 ND vs. PCD** ^c^ 0.08 WCD vs. PCD ^c^
Stroke history	17 (6.2%)	6 (9.1%)	3 (13.0%)	0.40ND vs. WCD ^c^ 0.21 ND vs. PCD ^c^ 0.58 WCD vs. PCD ^c^
Occluded vessel (*n*, %) ICA	50 (18.2%)	11 (16.7%)	3 (13.0%)	0.77 ND vs. WCD ^c^ 0.54 ND vs. PCD ^c^ 0.68 WCD vs. PCD ^c^
MCA M1/M2	173 (62.9%)	45 (68.1%)	15 (65.2%)	0.13 ND vs. WCD ^c^ 0.52 ND vs. PCD ^c^ 0.77 WCD vs. PCD ^c^
Tandem ICA/MCA	14 (5.1%)	6 (9.1%)	4 (17.4%)	0.21 ND vs. WCD ^c^ **0.02 ND vs. PCD** ^c^ 0.28 WCD vs. PCD ^c^
BA/PCA	17 (6.2%)	3 (4.6%)	(1 (4.3%)	0.61 ND vs. WCD ^c^ 0.72 ND vs. PCD ^c^ 0.97 WCD vs. PCD ^c^

Statistical significance marked in bold. NIHSS—National Institutes of Health Stroke Scale, PAD—peripheral artery disease, ICA—internal carotid artery, MCA—middle cerebral artery, BA—basilar artery, PCA—posterior cerebral artery. Statistical tests used: ^a^ Student’s *t*-test, ^b^ Mann–Whitney test, ^c^ Chi-squared Pearson’s test.

**Table 2:** Procedure times and procedural results.

Characteristic	Non-Diabetic Group (ND) (*n* = 275)	Well-Controlled Diabetic Group (WCD) (*n* = 66)	Poorly Controlled Diabetic Group (PCD) (*n* = 23)	*p* Value
Times in min (mean ± SD) Door-to-picture	21.7 ± 10.7	20.9 ± 9.8	22.4 ± 15.3	0.84 ND vs. WCD ^a^ 0.79 ND vs. PCD ^a^ 0.86 WCD vs. PCD ^a^
Door-to-groin	76.8 ± 19.4	82.7 ± 25.1	80.5 ±22.0	0.78 ND vs. WCD ^a^ 0.64 ND vs. PCD ^a^ 0.82 WCD vs. PCD ^a^
Onset-to-groin	215 ± 68.9	194.5 ± 79.7	222.5 ± 60.6	0.49 ND vs. WCD ^a^ 0.89 ND vs. PCD ^a^ 0.38 WCD vs. PCD ^a^
MT procedure time	76.0 ± 41.2	69.8 ± 34.6	68.5 ± 32.3	0.91 ND vs. WCD ^a^ 0.88 ND vs. PCD ^a^ 0.93 WCD vs. PCD ^a^
mTICI after intervention (*n*, %) Successful (TICI 2b-3)	219 (79.6%)	54 (81.8%)	20 (86.9%)	0.73 ND vs. WCD ^b^ 0.78 ND vs. PCD ^b^ 0.80 WCD vs. PCD
Unsuccessful (TICI 0-2a)	56 (20.4%)	12 (18.2%)	3 (13.1%)	0.75 ND vs. WCD ^b^ 0.82 ND vs. PCD ^b^ 0.89 WCD vs. PCD ^b^
Complications (*n*, %) sICH	21 (7.6%)	4 (6.0%)	5 (21.7%)	0.74 ND vs. WCD ^b^ **0.038 ND vs. PCD** ^b^ **0.046 WCD vs. PCD** ^b^
Groin hematoma	29 (10.5%)	8 (12.1%)	4 (17.4%)	0.71 ND vs. WCD ^b^ 0.31 ND vs. PCD ^b^ 0.52 WCD vs. PCD ^b^

Statistical significance marked in bold. sICH—symptomatic intracranial hemorrhage. Statistical tests used: ^a^ Mann–Whitney test, ^b^ Chi-squared Pearson’s test.

## Data Availability

The data presented in this study are available upon reasonable request from the corresponding author.
